# Driving gut microbiota enterotypes through host genetics

**DOI:** 10.1186/s40168-024-01827-8

**Published:** 2024-06-28

**Authors:** Catherine Larzul, Jordi Estellé, Marion Borey, Fany Blanc, Gaëtan Lemonnier, Yvon Billon, Mamadou Gabou Thiam, Benoît Quinquis, Nathalie Galleron, Deborah Jardet, Jérôme Lecardonnel, Florian Plaza Oñate, Claire Rogel-Gaillard

**Affiliations:** 1 GenPhySE, Université de Toulouse, INRAE, ENVT, Castanet Tolosan, 31326 France; 2 Université Paris-Saclay, INRAE, AgroParisTech, GABI, Jouy-en-Josas, 78350 France; 3 INRAE, GenESI, Surgères, 17700 France; 4 Université Paris-Saclay, INRAE, MGP, Jouy-en-Josas, 78350 France

**Keywords:** Gut microbiota, Genetics, Genetic selection, Enterotype, Holobiont, Metagenomics, Heritability, Pig

## Abstract

**Background:**

Population stratification based on interindividual variability in gut microbiota composition has revealed the existence of several ecotypes named enterotypes in humans and various animal species. Enterotypes are often associated with environmental factors including diet, but knowledge of the role of host genetics remains scarce. Moreover, enterotypes harbor functionalities likely associated with varying abilities and susceptibilities of their host. Previously, we showed that under controlled conditions, 60-day-old pig populations consistently split into two enterotypes with either *Prevotella* and *Mitsuokella* (PM enterotype) or *Ruminococcus* and *Treponema* (RT enterotype) as keystone taxa. Here, our aim was to rely on pig as a model to study the influence of host genetics to assemble enterotypes, and to provide clues on enterotype functional differences and their links with growth traits.

**Results:**

We established two pig lines contrasted for abundances of the genera pairs specifying each enterotype at 60 days of age and assessed them for fecal microbiota composition and growth throughout three consecutive generations. Response to selection across three generations revealed, per line, an increase in the prevalence of the selected enterotype and in the average relative abundances of directly and indirectly selected bacterial genera. The PM enterotype was found less diverse than the RT enterotype but more efficient for piglet growth during the post-weaning period. Shotgun metagenomics revealed differentially abundant bacterial species between the two enterotypes. By using the KEGG Orthology database, we show that functions related to starch degradation and polysaccharide metabolism are enriched in the PM enterotype, whereas functions related to general nucleoside transport and peptide/nickel transport are enriched in the RT enterotype. Our results also suggest that the PM and RT enterotypes might differ in the metabolism of valine, leucin, and isoleucine, favoring their biosynthesis and degradation, respectively.

**Conclusion:**

We experimentally demonstrated that enterotypes are functional ecosystems that can be selected as a whole by exerting pressure on the host genetics. We also highlight that holobionts should be considered as units of selection in breeding programs. These results pave the way for a holistic use of host genetics, microbiota diversity, and enterotype functionalities to understand holobiont shaping and adaptation.

Video Abstract

**Supplementary Information:**

The online version contains supplementary material available at 10.1186/s40168-024-01827-8.

## Background

A holobiont is defined as a living organism that embodies a host and its microbiota as a fully fledged biological entity [1]. A holobiont acts as a biological system that relies on complex and continual host-microbiota interactions. This new biological scale has paved the way towards a new field of research referred to as hologenomics that aims at integrating the genomic features of both the host and its microbiota [2, 3]. In humans and animals, the gut microbiota displays high inter-individual variability and determining the main drivers of this diversity remains an open question. It is acknowledged that the composition of an individual’s gut microbiota results from multiple factors including the environmental conditions at birth, the diet, age, genetics, sex, or medication administered [4]. A wide range of studies has already highlighted the influence of the host genetics on shaping the gut microbiota: first by the analysis of twin pairs in humans [5], and later with genome-wide association studies in humans [6, 7], mice [8], or pigs [9]. In addition, based on the estimates of microbiability, which is defined as the proportion of the phenotypic variance explained by microbiota variations in livestock, and of heritability, it has been established that microbiota can have strong links with host phenotypes and genotypes [10, 11]. In parallel, the comparison between intestinal microbiota catalogs has revealed limited sharing at the gene level across host species [12–15], which is in line with a tight co-evolution between the host and its microbiota during animal speciation that relies partly on adaptation to changes in diets [16]. In spite of all these results and clues on the links between a host’s genetics and its microbiota, in vivo animal models, which could confirm that the gut microbiota composition can be oriented by genetic selection directly exerted on the host, are still lacking.

**Fig. 1:**
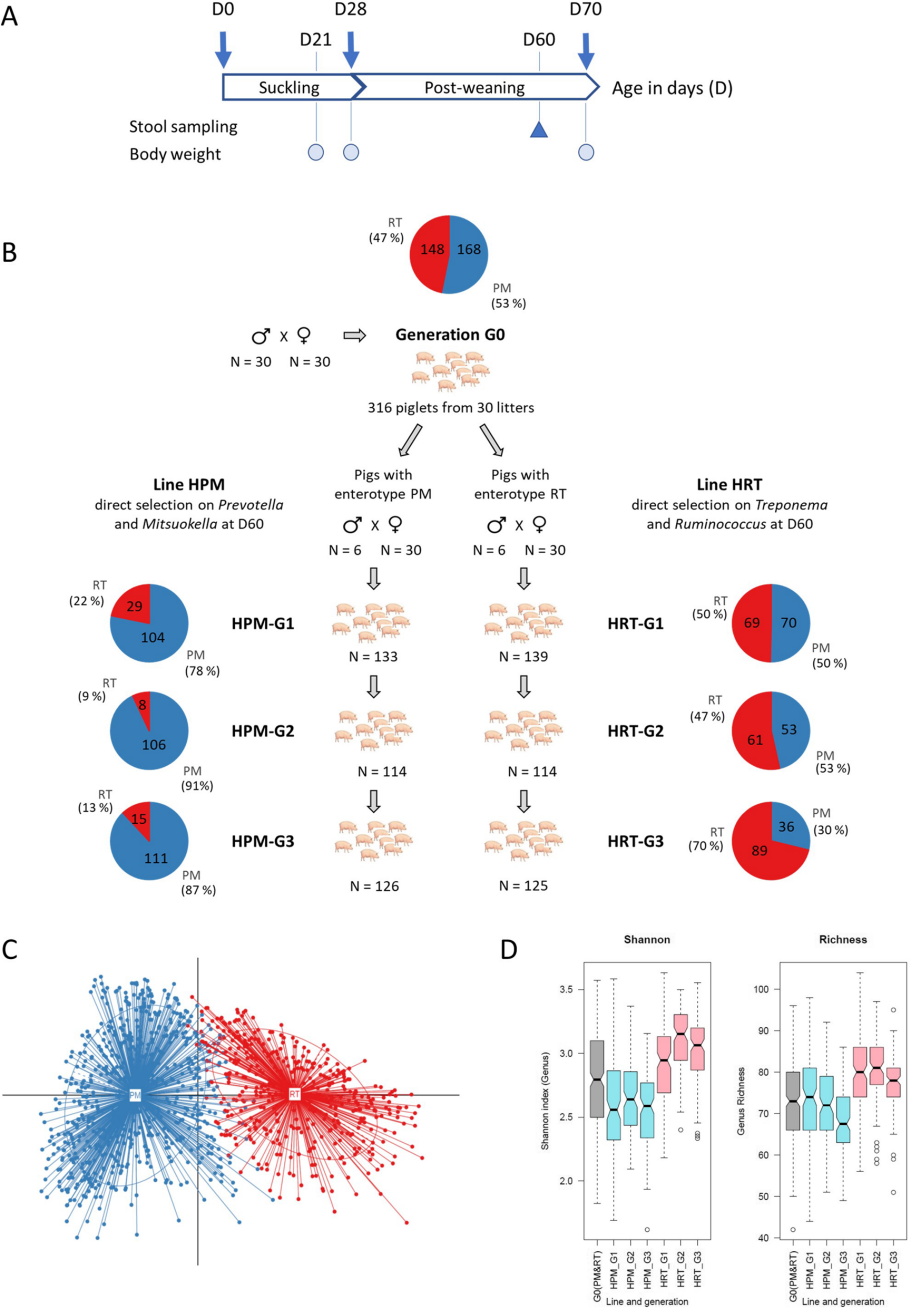
Animal protocol and stratification of the Large White pigs according to their enterotypes PM (*Prevotella*-*Mitsuokella*) or RT (*Ruminococcus*-*Treponema*). **A** Timing for stool sampling (blue triangle) and body weight records (colored circles) from birth (D0) until the end of the post-weaning period (D70). Microbiota data was obtained in all animals by sequencing the 16S rRNA gene using DNA extracted from stools sampled at D60. For a subset of 30 G0 animals, whole-metagenome sequencing data was obtained at D60. **B** Selection strategy of the two pig lines HPM (High PM) and HRT (High RT) over three successive generations (G1 to G3). The G0 generation was not genetically selected and corresponds to the founding population obtained from 30 litters from 30 males crossed with 30 females. The generations G1 to G3 comprised 30 litters produced from 6 males and 30 females each. The number of piglets per generation and per pig line is indicated. The relative prevalence of the two enterotypes within each population is represented by pie charts, the blue and red sections representing the % of pigs with the PM or RT enterotype, respectively. The number of pigs for each enterotype is reported in the pie charts. **C** Enterotype distribution of the whole population of 1067 pigs (generation G0 to G3, two pig lines) into two groups that correspond to the enterotypes PM (blue) and RT (red). **D** Notched box plots showing the differences in alpha diversity (left: Shannon index, right: richness) according to generation and pig line

Previously, we have shown that, at 60 days of age (D60), Large White pig populations bred under the same controlled conditions stratify into two enterotypes according to their fecal microbiota [17]. These two enterotypes are characterized by an overabundance of either *Prevotella* and *Mitsuokella* (PM), or *Ruminococcus* and *Treponema* (RT). These four genera were central nodes in inferred ecological networks and were subsequently considered keystone taxa for shaping the two enterotypes. Notably, the fact that a taxon was very abundant did not necessarily mean that it played a key role in driving an enterotype. Indeed, whatever the enterotype, *Prevotella* was the most abundant genus while *Ruminococcus* had a low abundance in all pigs. In this report, our aims were to study whether the selection of pigs for the relative abundance of the bacterial genera (used as a phenotype), which define the enterotypes PM or RT, is effective in orienting the composition of the gut microbiota of offspring, and to deepen the analysis of taxonomic and functional contrasts between the two enterotypes. For this study, we produced two pig lines referred to as HPM (high prevalence of PM) and HRT (high prevalence of RT) and monitored the response to selection across three generations (Fig. 1).

## Methods

### Animal farming and management, phenotyping, and biological sampling

The experiment was conducted on the INRAE experimental farm at le Magneraud (GenESI, Pig Phenotyping and Innovative Breeding Facility, 10.15454/1.5572415481185847E12).

Male and female piglets were weaned at the age of 28 days. At weaning, piglets from two to three different litters from the same line were mixed in post-weaning pens of 20 to 24 individuals. They received food and water ad libitum. The starter diet (18.6% protein and 10.8 MJ/kg net energy (NE) on a dry matter basis) was given during the last week before and the first 2 weeks after weaning, and the weaner diet (17.5% protein and 10.0 MJ/kg NE) was given from the second week after weaning. The piglets were transferred to growing-finishing pens at 10 weeks of age in pens of 10 to 12 individuals from the same post-weaning pen. All piglets were weighed at birth, at weaning, and at the end of the post-weaning period at around 70 days of age. Average daily gain was estimated for the post-weaning period from 28 to 70 days of age (ADG_postweaning). In total, 1067 animals were included in the study (Fig. 1B), comprising 316 piglets for the basal G0 population, 272 piglets for the G1 generation (133 HPM and 139 HRT), 228 piglets for the G2 generation (114 HPM and 114 HRT), and 251 piglets for the G3 generation (126 HPM and 125 HRT).

Stool samples were collected at 60 days of age directly from the rectal ampulla, snap-frozen in liquid nitrogen (approximately 200 mg per cryotube), and stored at − 80 °C until use for microbial DNA extraction. All biological samples were stored at the Biological Resources Center of the @BRIDGe core facility that is a member of the CRB-Anim infrastructure (10.15454/1.5613785622827378E12, CRB-Anim, INRA, 2018. Biological Resource Centers for domestic animals of AgroBRC).

### Selection experiment

For the G0 basal population, 30 Large White sows bred on the INRAE experimental farm were each inseminated once with semen from 30 Large White boars. The design was chosen such that a G0 generation was produced with maximal genetic diversity and with animals as lowly related as possible. Animals were selected on the relative abundance of either *Prevotella* and *Mitsuokella* or *Treponema* and *Ruminococcus *at 60 days of age, measured from 16S rRNA gene sequencing of fecal DNA, after precorrection for batch effect. Relative abundance of *Prevotella* was computed as the sum of *Prevotella_9*, *Prevotella_7*, and *Prevotella*, and relative abundance of *Ruminococcus * as the sum of *Ruminococcus*, *Ruminococcus_gnavus_group*, *Ruminococcus_torques_group*, and *Ruminococcus_gauvreauii_group*. At each generation, the male reproducers were selected on a family basis with only one boar per sire and using a mass selection approach. For the first generation of selection only, females were selected on a litter basis, with one or two females selected in the same litter for each line. This first step was designed to limit founders’ maternal effects in the first generation of selection. For the two other generations of selection, females were selected regardless of their family. At each generation, six boars and 30 females were selected as reproducers. The males and females with the highest abundances of *Prevotella* were selected for the HPM line and males and females with the highest abundances of *Treponema* were selected for the HRT line. At each generation, for the HPM line, male piglets were ranked intra-sire family according to their relative abundance of *Prevotella.* Among the top three male piglets per sire, the one with the highest relative abundance of *Mitsuokella* was selected as a future reproducer. For the HRT line, male piglets were ranked intra-sire family according to their relative abundance of *Treponema*, and among the top three male piglets per sire, the one with the highest relative abundance of *Ruminococcus* was selected as a future reproducer. Females were ranked within the line on *Prevotella* abundance for the HPM line and on *Treponema* for the HRT line. Males and females were mated according to their respective lines avoiding fullsib-halfsib matings.

### 16S rRNA sequencing and analysis

For 16S rRNA gene sequencing, DNA extraction was performed as previously described [18]*.* In brief, 200 mg of frozen fecal sample were resuspended with 250 μL of guanidine thiocyanate buffer (4 M guanidine thiocyanate–0.1 M Tris, pH 7.5), 40 μL of 10% N-lauroyl sarcosine–0.1 M phosphate buffer (pH 8.0), and 500 μL of 5% N-lauroyl sarcosine; the mixture was then incubated at 70 °C for 1 h. After the addition of one volume of 0.1-mm-diameter silica beads (Sigma), tubes were shaken for 10 min at the maximum speed on a Vibrobroyeur MM200 (Retsch, Germany). After shaking, the tubes were centrifuged at 20,000 g for 5 min at 4 °C. After recovery of the supernatant, 30 μL of Proteinase K (Chemagic STAR DNA BTS kit, Perkin Elmer, USA) were added; samples were incubated for 10 min at 70 °C at 250 rpm in a Multi-Therm shaker (Benchmark Scientific, USA) and then for 5 min at 95 °C for enzyme inactivation. After centrifugation at 20,000 g for 5 min at 4 °C, DNA extraction was performed on the supernatant using the Chemagic STAR DNA BTS kit (Perkin Elmer, USA) and the Chemagic STAR platform (Hamilton, Perkin Elmer, USA), according to the manufacturer’s instructions.

Amplicon libraries of the V3–V4 region of the 16S rRNA gene were constructed; amplification was performed using the PCR1F_343 (5′-CTTTCCCTACACGACGCTCTTCCGATCTACGGRAGGCAGCAG-3′) and PCR1R_784 (5′-GGAGTTCAGACGTGTGCTCTTCCGATCTTACCAGGGTATCTAATCCT-3′) primers following the Illumina 16S rRNA metagenomic sequencing library preparation protocol. Paired-end sequencing of the pooled library was performed on an Illumina MiSeq platform (Illumina Inc., San Diego, CA, USA) using the MiSeq Reagent kit v3 (2 × 300 cycles, Illumina Inc., San Diego, CA, USA), as previously described [18]. FastQ files were generated after the run was completed (MiSeq Reporter software, Illumina, USA).

We used DADA2 [19] to process reads into an amplicon sequence variant (ASV) table, with an approach that follows the authors’ recommendations in their “Big Data” workflow. In brief, for each MiSeq run, we performed separately the quality filtering, denoising pair-end merging, and amplicon variant calling steps. Briefly, for each run, primers were trimmed from read pairs by using cutadapt [20] and a quality filtering was performed on reads by using DADA2 filterAndTrim function with options truncF = 235, truncR = 225, and truncQ = 5. ASV unique sequences and corresponding counts per sample were inferred for each run with the DADA2 algorithm after learning the error distribution with the learnErrors function. This step included the merging of both read pairs and the removal of chimeras. Finally, the ASV tables from each run were merged based on ASV sequences, and the annotation of each ASV assigned in DADA2 by using the SILVA database (version 138) [21]. Based on the sequencing depth and the rarefaction curves, we rarefied counts for subsequent analyses at 7000 counts per sample. Alpha diversity was measured through microbial richness (that is, the number of taxa present in each sample after rarefaction) and the Shannon index estimated in vegan R package [22].

Enterotype categorization was done in R and was based on Jensen–Shannon divergence (JSD) distance measured from the rarefied genus abundance table as recommended by [23]. In brief, we first determined the optimal number of distinct clusters after partitioning the microbiota around medoids with all samples in each generation, and then confirmed if, as previously reported in 60-day-old pigs, the fecal microbiota was also divided into two enterotypes. Each individual was finally affiliated to its corresponding enterotype and the clustering was represented on a PCoA.

For the analysis of contrasts between the two enterotypes from piglets of the G0 population, we carried out the clustering process over 100 iterations and further considered only animals that never changed group (Figure S1). This process prevented us from including piglets that would have been randomly misclassified, and thus allowed us to confidently consider piglets that were good representatives of each enterotype. These animal subsets were used to deepen enterotype comparison and to select 15 females per enterotype for shotgun metagenomic sequencing.

### Statistics for analysis of genetic parameters and response to selection

All host genetic analyses were carried out using 16S rRNA sequencing data. Only relative abundances of genera representing more than 0.1% of the total were kept for genetic analyses. Thus, we performed genetic analyses on 64 relative abundances of bacterial genera, two diversity indexes (Shannon and richness) and the two relative abundances of the sum of *Prevotella* and the sum of *Ruminococcus* used for selection. All relative abundances were normalized with a log-ratio transformation, after adding 0.01 to remove 0 values.

For the genetic analysis, the fixed effects of batch and sex were included in the model. The random effects of common litter and animal were also included in the model. Heritability and common litter effect were first estimated with a one-trait animal model. All ancestors of the recorded animals up to five generations from the G0 animals were taken into account to build the additive relationship matrix for 4551 individuals. The estimation of genetic parameters was performed with the VCE6 software [24, 25].

The responses to selection were estimated by the differences between the two lines within generation, the data from the HPM line being used as baselines. For each parameter, we compared HPM-HRT differences within a generation over three generations. The analysis was carried out on diversity indexes, growth performances, and genera with less than 50% zero values (*N* = 74) including the 64 genera mentioned above. The relative abundances were log-transformed after adding 0.01 to remove zero values. The differences were obtained with the GLM procedure (SAS9.4). The model included the effect of sex, batch nested intra-generation, and a generation × line interaction. The level of significance for the differences between the two lines within a generation was estimated by the contrast option. An adjusted level of significance was also estimated with the lsmeans option, with a Tukey adjustment. The analysis was carried out on standardized variables, centered-standardized variables, and ranked variables. In order to illustrate the response to selection, a ranking was performed on the HPM-HRT differences observed between the two lines in the G3 generation for the standardized variables, and differences were then plotted.

### Shotgun metagenomics and analysis

DNA extraction was performed by the SAMBO platform located at INRAE MetaGenoPolis. Fecal DNA was extracted following the SOP 07 V2 H from [26, 27]. The DNA preparation was subjected to quality control using Qubit Fluorometric (ThermoFisher Scientific, Waltham, USA) and qualified using DNA size profiling on a Fragment Analyzer instrument (Agilent Technologies, Santa Clara, USA).

Sequencing was performed by the MetaQuant platform located at INRAE MetaGenoPolis. Three micrograms of high molecular weight DNA (> 10 kbp) were used to build sequencing libraries. Shearing of DNA into fragments of approximately 150 bp was performed using an ultrasonicator (Covaris, Woburn, USA) and DNA fragment library construction was performed using the Ion Plus Fragment Library and Ion Xpress Barcode Adapters Kits (ThermoFisher Scientific, Waltham, USA). Purified and amplified DNA fragment libraries were sequenced using the Ion Proton Sequencer (ThermoFisher Scientific, Waltham, USA). The raw sequences are available through the project PRJEB60032 on the EMBL-EBI’s European Nucleotide Archive (ENA accessions ERS14678539 to ERS14678568).

Quality control was performed with AlienTrimmer [28]: (1) sequencing adapters were removed, (2) low-quality reads were trimmed or discarded, and (3) reads that were too short (< 60 bp) were discarded. Then, reads mapped to the pig reference genome (Sscrofa11.1 GCA_000003025.6) with bowtie2 [29] were removed. Finally, 18 M high-quality reads were randomly selected in each sample with fastq-sample [30].

The gene abundance table was generated with the METEOR software suite [31]. First, selected high-quality reads were mapped with bowtie2 [29] to a gene catalog representative of the pig gut microbiota [32], comprising 9.3 million genes. Alignments with nucleotide identity < 95% were discarded and gene counts were computed with a two-step procedure previously described that handles multi-mapped reads [33]. Finally, raw gene counts were normalized according to gene length.

Using MSPminer [34], the gene catalogue was previously organized into 1523 MetaGenomic species (MGS), which are clusters of co-abundant genes corresponding to the same microbial species. The abundance of a MGS in a sample was defined as the mean abundance of its 100 marker genes (i.e., species-specific core genes that correlate most with each other). If less than 10% of the marker genes were found in a sample, the abundance of the MGS was considered null. Abundances at higher taxonomic ranks were computed as the sum of the MGS that belong to a given taxon. Taxonomic annotation of the MGS was carried out with GTDB-Tk [35] based on Genome Release 07-RS207.

KEGG Orthologs (KOs) were assigned to genes in the catalog with KofamScan based on the KEGG 102 database. KO abundances were computed by summing the abundance of genes assigned to the same KO.

Differentially abundant MGS or functional modules were searched for by using Wilcoxon–Mann–Whitney tests. False discovery rate (FDR) was controlled by correcting *p*-values for multiple testing with the Benjamini–Hochberg procedure. Effect size was estimated using the Cliff’s Delta statistic (CD) with the package effsize v0.7.4. Unless stated otherwise, features with corrected *p*-values (*q*-values) below 0.1 and a magnitude of the effect size |CD|> 0.7 were reported in Supplementary Tables (Table S1, Table S2).

The pathway enrichment analyses were performed on the lists of differentially abundant KOs by using the clusterProfiler R package [36]. In this package, we used the enrichKEGG function, with the hypergeometric distribution in over-representation analysis to calculate enrichment *p*-values, and used the list of KOs present in the differential analyses as background genes in the tests (significance: *p* < 0.05, Table S3).

## Results

### Stratification of the pig population into two contrasted gut enterotypes at D60

The founding G0 population comprised 316 60-day-old Large White piglets from 30 lowly related families, and their fecal microbiota was characterized by 16S rRNA gene sequencing and analysis of amplicon sequence variants (ASVs) (Fig. 1A).

**Fig. 2:**
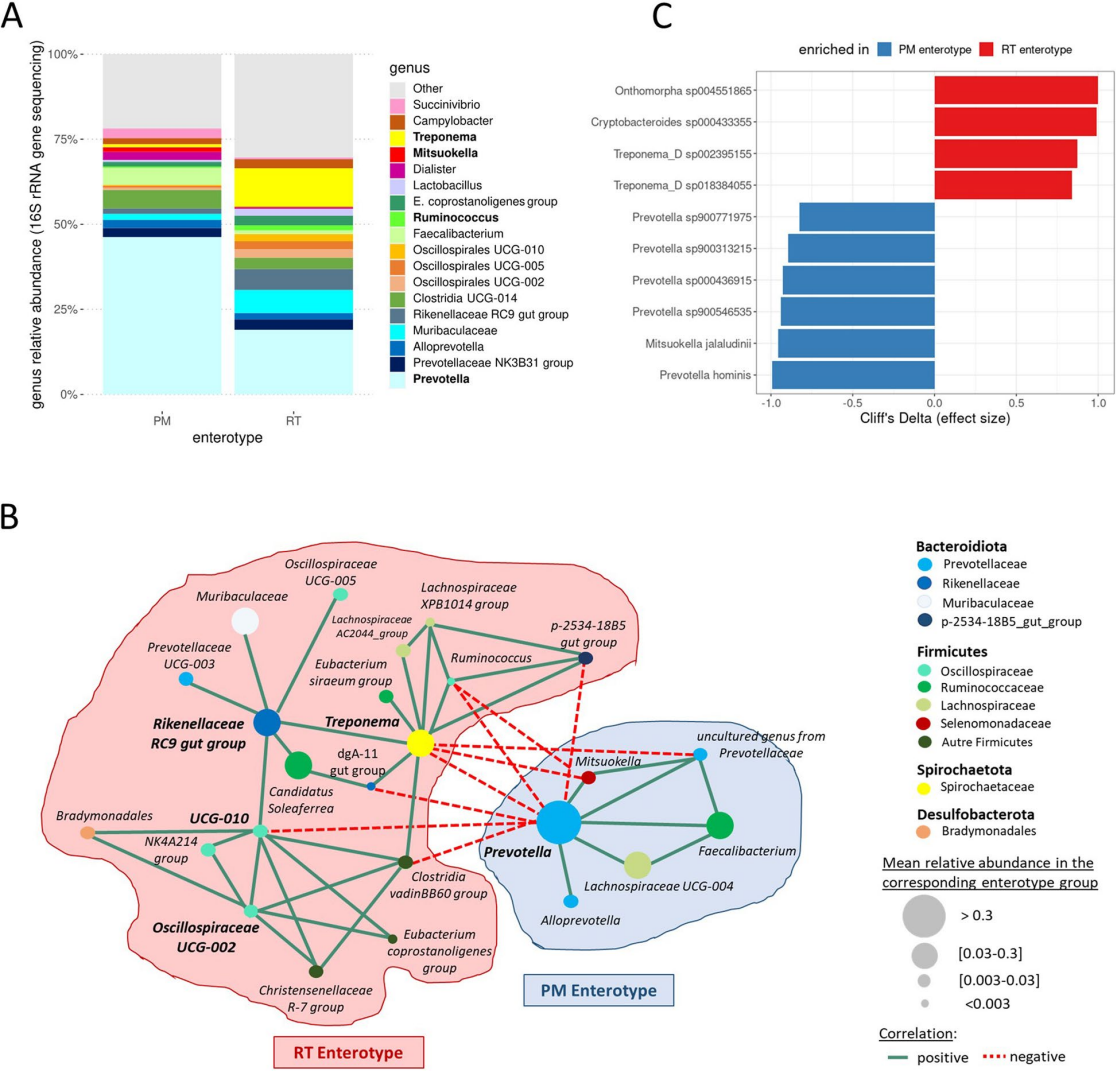
Main taxonomic and functional differences between the two enterotypes that were characterized on animals from the G0 basal population. **A** Average relative abundances of the main bacterial genera for the two pig groups harboring either PM (142 piglets) or RT (87 piglets) enterotypes based on 16S rRNA sequencing data. This analysis was narrowed down to the piglets with an unchanged enterotype across multiple repeats of the clustering process. **B** Co-abundance networks based on the most abundant and differentially abundant genera between the two enterotypes defined on the G0 population at D60, using 16S rRNA sequencing data. **C** Most contrasted MetaGenomic species (MGS) identified based on shotgun metagenomics data between two subsets of 15 females representative of each enterotype at G0. Effect size was estimated using the Cliff’s Delta statistic. Blue and red bars correspond to MGS enriched in PM and RT animals, respectively

Consistent with our previous study [17], the G0 population was stratified into two groups according to their fecal microbiota. One group was characterized by an overabundance of the *Prevotella* and *Mitsuokella* genera (PM enterotype, 168 piglets), and the other by an overabundance of the *Ruminococcus* and *Treponema* genera (RT enterotype, 148 piglets) (Fig. 1B). We analyzed the differences between the two enterotypes (Fig. 2) on two subsets of piglets that were consistently assigned to the same enterotype across 100 classification repeats (Figure S1). Although there was considerable variation in taxa abundance within each enterotype (Figure S2), the average taxonomic composition of each enterotype at the genus level showed the dominance of *Prevotella* for both enterotypes and a higher abundance of *Treponema* for the RT enterotype compared to *Ruminococcus* (Fig. 2A). This is the reason why, at each generation, the reproducing males and females for the HRT line ranked first on *Treponema* and second on *Ruminococcus*. The co-abundance network (Fig. 2B) confirmed that genera that were directly targeted for divergent selection are major hubs in the network, as in previous results [17]. Thus, the directly selected genera interact with many other taxa that need to be taken into account as indirectly selected in the selection process.

To carry out high-resolution taxonomic profiling and obtain the first clues on functional differences between the two enterotypes, shotgun metagenomics was performed on a subset of samples from 30 females from the G0 population, which was split into two groups, each group being a good representative of each enterotype (15 PM and 15 RT pigs) (Figure S1). We selected animals of the same sex to avoid a confounding factor that cannot be controlled with a limited number of animals. These analyses confirmed that both enterotypes were dominated by the genus *Prevotella*, although the latter was significantly more abundant in the PM animals (PM: 44.9% ± 8.3%; RT: 25.7% ± 10.0%). We identified *P. hominis* and several uncharacterized *Prevotella* bacteria as the main contributing species (Fig. 2C, Figure S3, and Table S1). In the PM animals, other genera were also enriched, including *Mitsuokella* (PM: 1.2% ± 1.2%; RT: 0.4% ± 0.5%) that was represented only by the species *M. jalaludinii*. As expected, the RT enterotype was enriched in *Ruminococcus* (RT: 1.3% ± 0.8%; PM: 0.5% ± 0.5%) and *Treponema* (RT: 8.7% ± 6.5%; PM: 1.2% ± 1.4%), the latter being mainly represented by two uncharacterized species (*T. sp002395155* and *T. sp018384055*). Notably, the *Rikenellaceae* *RC9* gut group was abundant and enriched in the RT animals (RT: 5.0% ± 2.3%; PM: 1.8% ± 1.1%) and consisted of two unknown species with temporary names (*Cryptobacteroides sp000433355* and *Onthomorpha sp004551865*) (Fig. 2C, Figure S3, and Table S1). Consistent with the average relative abundance of genera in each enterotype and the co-abundance network, this analysis suggested that combined with *Treponema*, the *Rikenellaceae RC9* gut group might better specify the RT enterotype than the *Ruminococcus* group (Fig. 2B). Using the KEGG Orthology database, we searched for differentially abundant functional orthologs (KOs) between the two enterotypes (Table S2). Strikingly, KOs related to starch degradation and polysaccharide metabolism were among the most enriched features in the PM enterotype. In the RT enterotype, the enriched KOs were different and included general nucleoside transport and peptide/nickel transport. The top-4 enriched pathways in the PM enterotype were related to the overall biosynthesis of amino acids and more specifically to the biosynthesis of phenylalanine, tyrosine, tryptophan, valine, leucine and isoleucine. By contrast, degradation of valine, leucine, and isoleucine was in the top-4 enriched pathways in the RT enterotype, which shows that both enterotypes harbor different functionalities relating to the same amino acid metabolism. In the RT enterotype, the three other top-4 functions were ABC transporter, metabolism of carbon, and metabolism of methane (Table S2 and Table S3). These contrasts are likely linked to differences in alpha diversity between enterotypes (Fig. 1D), with a potentially higher functional richness in the RT animals that needs deeper investigation.

### Selection of two divergent pig lines with gut microbiota enriched in bacterial genera specifying each enterotype

In this study, we hypothesized that host genetic selection directly targeting the four genera predicted to specify the two enterotypes could successfully drive the ecological structure of the gut microbiota. These four genera were chosen as direct targets for genetic selection as they were previously predicted as keystone taxa based on the analysis of co-abundance networks [17]. Within each group, animals were ranked by decreasing abundance of the two keystone taxa of their respective enterotype. Using this strategy, the males and females that were the most representative of each enterotype were chosen as founders of either the HRT or the HPM line. Under the same farming conditions, we produced three successive generations per line, by mating the best-ranked boars (*n* = 6) and females (*n* = 30) (Fig. 1B). After combining the whole cohort of 1067 pigs (G0, G1, G2, and G3 generations), enterotype clustering based on the D60 fecal microbiota composition confirmed a robust stratification into the same two enterotypes, without revealing any additional animal clusters that might have emerged during the genetic selection process (Fig. 1C). In agreement with our hypothesis on the role of the host genetics, throughout the three successive generations, the prevalence of the PM enterotype dramatically increased in the HPM pig line (from 53% in G0 up to 87% in HPM-G3), while that of the RT enterotype dramatically increased in the HRT line (from 47% in G0 up to 70% in HRT-G3). Notably, alpha diversity indexes (Shannon index and richness) were significantly higher in the HRT pig line than in the HPM pig line (Fig. 1D), which is consistent with the results of our previous study [17], and in agreement with those of reports on a lower alpha diversity of human enterotypes with *Prevotella* [37].

### Significant influence of host genetics on microbiota composition and enterotype assembly, and correlations with body weight traits

**Fig. 3:**
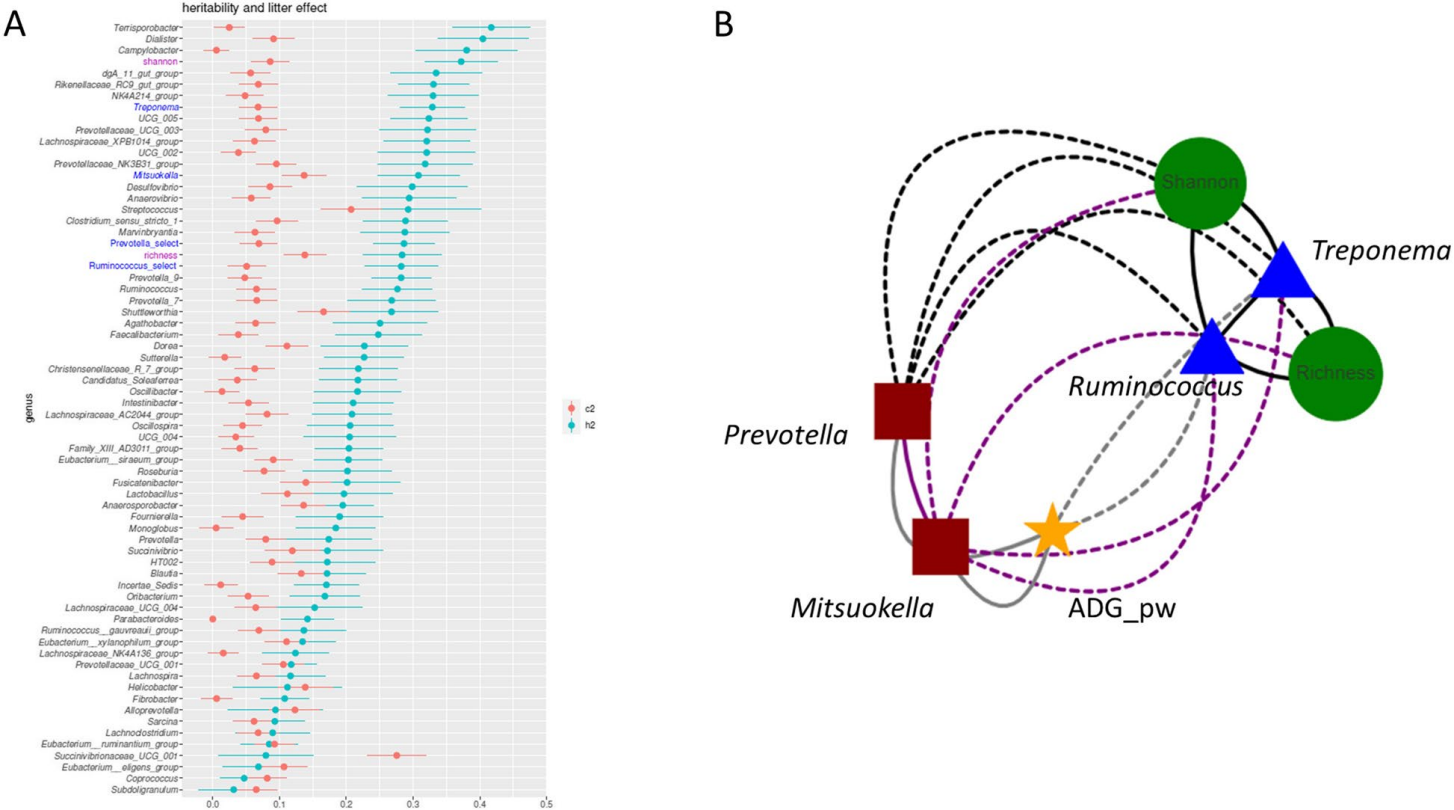
Genetic parameters of fecal microbiota composition and body weight phenotypes at D60. **A** Heritability estimates (h^2^ blue dots) and litter effect (c^2^ pink dots) with their standard error (lines) for 64 gut microbiota genera and 2 diversity indexes (genera) based on 16S rRNA sequencing data. Selected genera are written in dark blue (**B**). Genetic correlation between genera under selection in the HPM line (red squares) or the HRT line (blue triangles), diversity indexes (green disk), and post-weaning growth rate (ADG_pw, yellow star). Negative correlations are marked with dashed lines. Black lines represent correlations for which the absolute value is higher than 0.9, purple lines represent correlations with absolute values between 0.6 and 0.9 and gray lines correlations with absolute values between 0.3 and 0.5

Using the whole population, the estimates of the heritability for the four genera under direct selection were all within the same range, around 0.3. Among the four genera, the estimate of the heritability was highest for *Treponema*, and lowest for *Ruminoccocus* (Fig. 3A). The heritability for enterotype value as a unique binary trait (PM/RT) was estimated at around 0.3, consistent with an increase in enterotype prevalence in HPM and HRT lines over generations (Fig. 1B). Considering the 64 analyzed genera, the estimates of heritability for the corresponding bacterial genera ranged from 0.14 to 0.4. Several studies have reported heritability estimates for the relative abundance of bacterial genera of the gut microbiota in pigs either younger or older than D60 [38–40]. Because of differences in the animal genetic background or the age of the pigs analyzed or of differences between the statistical models used to estimate the genetic variances, it is difficult to compare the heritability estimates between studies. However, in all these studies, several genera had moderate to high heritability estimates, which shows that the host genetics has an influence on microbiota composition. The effect of common litter environment was small for a majority of the genera (< 0.1). We observed that the largest litter effect was estimated for *Streptococcus* and an unknown genus of the family *Succinovibrionaceae* (> 0.2). These findings revealed that, in our study, the mother and associated perinatal environment at birth and during the suckling period had a much weaker influence than the host genetics in shaping piglet gut microbiota at D60.

Genetic correlations between the four genera under direct selection were very high (Fig. 3B). Consistent with the enterotype structure of the HPM and HRT lines, the correlations between the bacterial genera characterizing the same enterotype were positive and the correlations between bacterial genera characterizing different enterotypes were negative. These four genera were also moderately correlated (absolute values from 0.32 to 0.52, with a standard error around 0.12) with post-weaning average daily gain (heritability value of 0.2) either positively (*Prevotella* and *Mitsuokella*) or negatively (*Ruminococcus* and *Treponema)*, which illustrates the phenotypic links observed previously between enterotypes and growth [17, 41]. The positive correlations between post-weaning growth and PM enterotype could be associated with the significant enrichment of gene families that are involved in starch degradation, as revealed by shotgun metagenomics. The genetic correlations show a positive co-selection of co-abundant genera per pig line with either a lower or higher ability to body weight gain in the HRT or HPM lines, respectively. These results suggest a response to selection at the holobiont level, which results in an extended phenotype that combines the growth capacity of the host together with its associated favorable microbiota. Interestingly, the RT enterotype is richer than the PM enterotype (Fig. 2B) but less efficient for piglet growth during the post-weaning period.

### Increased differences in bacterial genera abundances and post-weaning body-weight gain between the HPM and HRT lines across three generations of selection

**Fig. 4:**
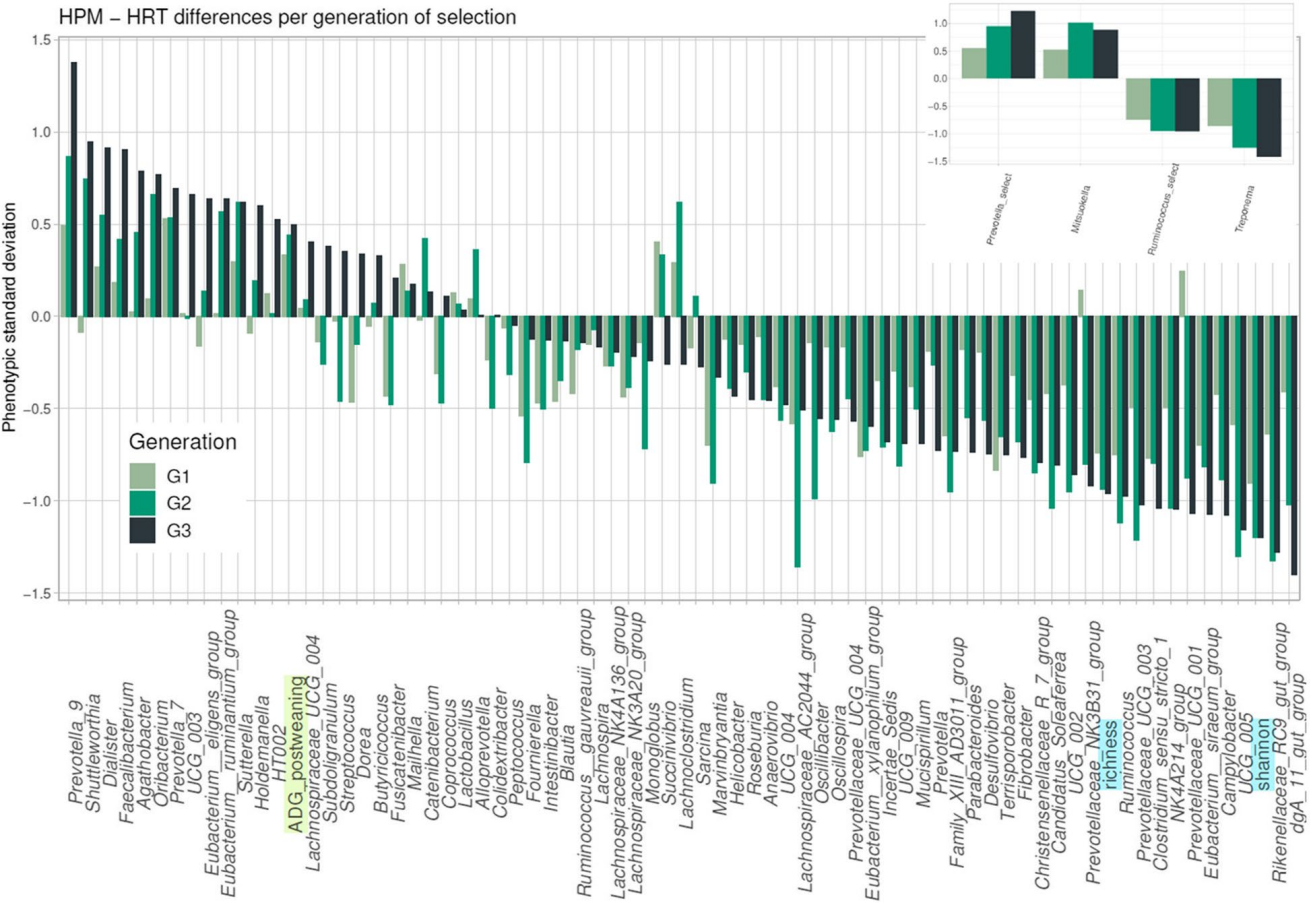
Responses to genetic selection oriented towards a high abundance of *Prevotella* and *Mitsuokella* or a high abundance of *Ruminococcus* and *Treponema* at D60 across three generations. The differences between the HPM and HRT lines for 74 genera, 2 diversity indexes (light blue highlighting), and one growth feature (ADG_postweaning: average daily gain during the post-weaning period, light green highlighting) are expressed in standard deviation. They are ranked from the largest positive difference (on the left) to the largest negative difference (on the right) at generation G3 of the selection. The differences are in pale green for G1, green for G2, and dark green for G3. Positive differences are associated with higher values in the HPM line whereas negative values are associated with higher values in the HRT line. Microbiota results are based on 16S rRNA sequencing data

The responses to selection were calculated as normalized differences between the HPM and HRT lines at each generation of selection for features such as bacterial genera, alpha diversity indexes (richness, Shannon), and body weight traits (Fig. 4). Considering the respective ratio of selected males (1/10) and selected females (1/2) within each line, selection is mainly driven by selection intensity on the sires. The differences between the HPM and HRT lines increased at each generation for *Prevotella* and *Treponema* and tended to be maintained between the G2 and G3 generations for *Ruminococcus* and *Mitsuokella*. *Prevotella* and *Treponema* are more abundant than the two other genera under direct selection and acted as main drivers of selection. For a few genera, the evolution trend was similar to that of either *Prevotella* or *Treponema*. For example, *Dialister*, *Faecalibacterium*, or *Agathobacter* were consistently higher in the HPM line than in the HRT line across the three generations of selection. The response to the selection of the *Rikenellaceae RC9* gut group had a similar evolutionary pattern than the *Ruminococcus or Treponema* groups. Considering its importance in enterotype representation combined with its high heritability estimate, this group should likely be considered as a valuable alternative or additional taxon for selection on enterotype. In general, the number of genera with a negative difference between the two lines in G3 is larger than the number of genera with positive differences, which is consistent with the PM animals having microbiota with a lower diversity. The differences between the HPM and HRT lines are in agreement with the co-abundance network that describes the two enterotypes (Fig. 2B). Thus, our selection experiment provides original data on the evolution of the microbiota as a biological ecosystem that responds to the genetic selection of its host; it would be nearly impossible to predict such an ecosystemic evolution by considering the genetic and phenotypic correlations between all genera.

We also included the calculation of the effect of selection on post-weaning growth (ADG_postweaning) since significant genetic correlations with microbiota components were estimated (Fig. 4). The contrasts between the two pig lines increased at each generation with greater post-weaning growth in the HPM line, confirming a combined selection for host and microbiota features during the selection process.

## Discussion

Stratification of human populations according to their gut microbiota composition has led to defined enterotypes that reduce the microbiota complexity to a limited number of ecosystems with different microbial diversities and functionalities. In spite of the initial controversies on their associated concept and reliability [42, 43], three enterotypes have been reported and further confirmed in humans, their key taxa being *Prevotella*, Firmicutes (including the genus *Ruminococcus*), and *Bacteroides* [23, 37]. Enterotypes have also been characterized in wild and domesticated animals (e.g., chimpanzees [44], wild mice [45], domesticated pigs [17, 41], African buffaloes [46]), which show that enterotypes are not exclusive of human microbiomes [37] and may reflect long-term host-microbiota interactions that accompany the coevolution of both the host species and its gut microbiota. This coevolution process may have resulted in a limited number of self-beneficiary ecotypes providing beneficial services to their hosts [47]. In such a “leash model” that was conceptualized within the ecology disciplinary field, it is predicted that the traits that benefit the host will also help bacteria to persist in the microbiome. Thus, investigating how host biology affects the microbiome might be as important as elucidating how symbionts affect their hosts since the benefits provided by microbes are typically by-products of microbe species that strive to be represented in the microbiome [47]. In line with this need to assess the host control on its microbiome, we report original data on the influence of the host genetics for shaping its gut microbiota and associated enterotypes in pigs, which raise issues for breeding programs that should consider the holobiont and not only the host.

Our results confirmed that populations of 60-day-old Large White piglets repeatedly and consistently stratify according to the two enterotypes PM and RT under shared and controlled environmental and feeding conditions. We experimentally demonstrated a significant influence of the host genetics by producing two pig lines that were selected throughout three generations for relative abundances of bacterial genera identified as key taxon drivers for assembling the enterotypes. Our data demonstrate that the factors that determine the enterotype composition, at least at this young age, are not predominantly linked with dietary habits and environmental constraints as usually stated [18, 46]. We focused on the age of 60 days since it corresponds to a reference time point in our first large-scale study reporting the pig enterotypes [17]. Under our experimental conditions, D60 is 32 days after weaning and 10 days before starting the fattening period until slaughter between 140 and 150 days of age. In pigs, the gut microbiota diversifies considerably after birth during the first 3 weeks with a dramatic shift before and after weaning [39, 48–50]. The age of 60 days is an interesting time point in pig life to study gut microbiota composition because it corresponds to the first step of microbiota maturation and stability [41].

The RT enterotype was found to be richer than the PM enterotype but less efficient for body weight gain measured on piglets from 28 to 70 days of age. The gut microbiota richness has been acknowledged as a robust indicator of gut health and resilience capacity [51]. Therefore, the RT enterotype might be more resilient than the PM enterotype to stressors or suboptimal farm conditions in spite of being less efficient for growth at early life stages. Our results challenge the general assumption that fast growth is linked to good health. Indeed, we suggest the need to investigate possible vulnerabilities and physiological tradeoffs in piglets that grow very fast at early life stages. The less diverse PM enterotype seems to be enriched in functional features that may favor piglet growth capacity, with functions relating to starch degradation and polysaccharide metabolism, overall biosynthesis of amino acids and more specifically to the biosynthesis of phenylalanine, tyrosine, tryptophan, valine, leucine, and isoleucine. Strikingly, the two enterotypes were found to have opposite functionalities for the metabolism of valine, leucine, and isoleucine, the PM enterotype being oriented towards their biosynthesis and the RT enterotype towards their degradation. As in our study, the *Prevotella* enterotype in humans was also characterized by the lowest alpha diversity and associated with high dietary fiber intake. This is particularly highly prevalent in individuals with non-Western and/or fiber-rich diets [18]. In both the PM enterotype in pigs and the *Prevotella* enterotype in humans, there is an enrichment in enzymes that degrade fibers.

Heritability estimates of around 0.3 were found for the four genera under direct selection. The same bacteria genera were reported with heritability estimates lower than 0.2 when measured on animals at around 300 days of age [38] or 110 days of age [39] or with null heritability estimates when measured on lactating piglets [40]. At older ages, some other genera were found with a h^2^ value of around 0.3, higher than our estimates (e.g., *Blautia*, *Paraprevotella*, *Roseburia*, *Streptococcus*, *Succinivibrio* [38], *Anaevibrio*, *Rikenellaceae RC9* gut group, *Dialister* [39]). These variations in heritability estimates for the same bacteria genera at different ages suggest an influence of the host genetics that may vary in importance throughout life but that is strong in pigs at D60 compared to younger ages. In suckling piglets, we hypothesize that it is too early to estimate genetic parameters since the dynamic process of microbiota diversification is just starting with likely strong daily inter-individual differences. Host genetics has been documented as a determinant of microbiomes in a wide range of animals from animal models to wild or livestock species and humans. Our results highlight the possible varying importance of the host genetics on gut microbiota composition throughout life and are relevant for making links with targeting strategic time windows for efficiently modulating the gut microbiota. Our hypothesis of a varying influence of the host genetics throughout life might help to reconcile divergent assumptions on the respective importance of the host genetics [5] and the environment [52] in shaping the gut microbiota.

We show that selecting for the relative abundance of bacterial genera that are identified as key drivers of enterotypes results in a selection at the whole ecosystem level, with increased contrasts at each pig selection generation for all genera identified as differentially abundant between the two enterotypes at generation G0. This highlights that enterotypes are consistent functional ecosystems that can be selected as a whole by exerting pressure on the host genetics. Since compared to the RT enterotype the PM enterotype is associated with faster growth at early life stages, it might be tempting to select for a higher prevalence of PM enterotype at D60. However, it might be important to preserve the microbiomes as flexible biodiversity throughout life for resilience at the animal population level. For instance, in African buffalos, two enterotypes have been reported to be driven by *Ruminococcaceae-UCG-005* or *Solibacillus* [46]. The *Ruminococcaceae-UCG-005*-driven enterotype is the richest and was found to be prevalent when the animals were submitted to resource-abundant dietary regimes, with limited beta diversity, while the *Sollibacillus*-driven enterotype is less rich and was found to be prevalent in restricted dietary conditions but with a high beta diversity. The environmental-based shift in enterotype prevalence is associated with increased beta diversities for the less diverse enterotype, thus contributing to maintaining the gamma diversity at the population level and favoring the recolonization via microbial sharing across hosts when the environment allows it [46]. In humans, it has been shown that individuals may change enterotypes throughout life [42]. It will be highly interesting to investigate the temporal dynamics of enterotypes in the same pigs and to study the impact of harboring the PM or RT enterotype at D60 for production, health, welfare, resilience, and longevity traits in the long term throughout life. It will also be important to investigate whether preserving the two enterotypes as complementary genetic resources is beneficial for resilience and sustainability at the pig population level.

During the last decade, holobionts have emerged as units of biological organizations that exhibit synergistic phenotypes that are subject to evolutionary forces [2, 53] with theoretical and research issues addressed to all life sciences, including biomedical, ecological, and agronomic sectors. Genetic variations between hologenomes may be due to changes in the host genome as well as in the genomes of symbiotic microbes, leading to a complex framework in which the host genome and microbiome forge networks of G(host) x G(Microbiome) interacting with the environment. Thus, holobionts may be considered as units of selection that combine host genetics provided at the host conception and gut microbiota that starts at birth and is dynamic throughout the whole life of its host. Considering holobionts in breeding programs is challenging. Our results clearly demonstrate that selecting components of the gut microbiota can be done with potential benefits for growth during the post-weaning period. However, targeting this benefit via genetic selection on the PM enterotype would potentially be associated with a less diverse gut microbiota. The intense breeding programs in pigs that are fed formula feeds that contain high levels of protein and energy may have already eroded the level of biodiversity of the gut microbiota. Clues on such biodiversity erosion are provided by an expanded gene catalog of the pig gut microbiome that includes data from wild boars [54]. This expanded catalog revealed that the alpha-diversity of the gut microbiota is higher in wild boars than in commercial Duroc pigs and that the gut microbiota is enriched in a number of pathways including amino-acid biosynthesis and metabolism, lipid, carbohydrate, and vitamin (B6, Biotin) metabolism, antibiotic biosynthesis [54]. However even if the microbiota diversity has decreased in commercial pigs, the existence of two enterotypes is preserved. In that respect, as discussed in human studies [18, 42, 43], there is a need to deepen research on whether enterotypes may be considered as relevant biomarkers for welfare, fitness, or risk assessment. Actually, the very limited number of enterotypes per host species (three in humans, and in our study two in pigs) is striking considering the high level of gut microbiota complexity and variabilities across individuals. The sole use of enterotype information may hide more subtle differences that are embedded within the enterotype. We may anticipate that elaborating breeding programs in livestock at the holobiont level will need to efficiently combine covariations between host genomes and their microbiome selection and modulation, in order to preserve the microbiome richness and diversity for resilient and sustainable livestock systems. The HPM and HRT divergent pig lines will potentially contribute to a better understanding of the combined impact of host genetics and gut microbiota on a range of phenotypes that are relevant for sustainable livestock systems, from growth and feed efficiency to health and welfare.

## Conclusion

In this paper, we provide a formal demonstration that direct host genetic selection for the relative abundance of a very limited number of bacterial genera that are identified as key taxa driving the two enterotypes PM and RT is effective throughout generations, and orientates the whole gut microbiota as a functional ecosystem, by indirectly selecting for interacting bacteria. This study was performed on 60-day-old piglets and additional experiments would be necessary to study the possible changes of enterotypes over animal’s life and variations in the impact of host genetics on the gut microbiota at ages older than 60 days. Nevertheless, our report highlights that holobionts may be considered as units of selection with intimate mechanisms between the host and its microbiota that need to be further investigated. Shotgun metagenomics have revealed some first clues to understanding the functional differences between pigs with the RT enterotype, which is more diverse, and those with the PM enterotype, which is more efficient for piglet growth during the post-weaning period. Overall, our results pave the way for future programs that aim at defining breeding goals at the holobiont level, which will favor livestock sustainability while both preserving host and microbiota biodiversity. In addition, this study raises issues that are shared by biomedicine and life sciences on how to optimize the holistic use of host genetics, gut microbiota diversity, and enterotype functionalities for assessing and predicting disease risks and traits of interest.

### Supplementary Information

Supplementary Material 1: Figure S1. Clustering of 60-day-old piglets from the G0 basal population into two enterotypes. (A) 313 G0 piglets comprising 86 piglets with varying enterotype classification during the clustering process over 100 iterations; (B) The subset of 227 piglets that do not change enterotype during the clustering process over 100 iterations. The subset of 15 females submitted to the shotgun metagenomics analysis were chosen from these two groups that are always categorized to the same enterotype.

Supplementary Material 2: Table S1. Differentially abundant MGS, Top 100 MetaGenomic Species significantly enriched in PM animals compared to RT and Top 100 MetaGenomic Species significantly enriched in RT animals compared to PM (Wilcoxon–Mann–Whitney test: FDR<0.1).

Supplementary Material 3: Table S2. Differentially enriched KO, Top 100 KOs significantly enriched in PM animals compared to RT and Top 100 KOs significantly enriched in RT animals compared to PM (Wilcoxon–Mann–Whitney test: FDR<0.1).

Supplementary Material 4: Table S3. Differentially enriched pathways, Enriched KO pathways in PM and RT enterotype metagenomes were obtained by using the enrichKEGG function (*p*<0.05) using the full lists of differentially abundant KOs in the clusterProfiler R package.

Supplementary Material 5: Figure S2. Individual variability of the taxonomic composition of each enterotype at the genus level on 60-day-old piglets from the G0 basal population. Only piglets that did not change enterotype during the classification process were represented on this figure. Barplots represent the relative abundance of the main genera observed in the communities (median relative abundance above 1.5 in at least one enterotype).

Supplementary Material 6: Figure S3. Differential abundances of a selection of MetaGenomics species (MGS) between PM and RT enterotypes. Abundance of the 12 most enriched MGS identified with shotgun metagenomics in either RT (A) or PM animals (B). Two subsets of 15 females are considered, blue and red boxplots corresponding to PM and RT animals respectively. Here, the abundance is the estimated genome sequencing coverage at similar sequencing depth.

## Data Availability

Data generated during the current study are available on public repositories. − Animals and phenotypes https://entrepot.recherche.data.gouv.fr/dataset.xhtml?persistentId=doi:10.57745/G06TTE. − Summary of fecal microbiota information https://entrepot.recherche.data.gouv.fr/dataset.xhtml?persistentId=doi:10.57745/P6JXS2. − Animal pedigrees https://entrepot.recherche.data.gouv.fr/dataset.xhtml?persistentId=doi:10.57745/UAPQQB. − The raw sequences are available through project PRJEB60032 on the EMBL-EBI’s European Nucleotide Archive: ENA accessions ERS14670635 to ERS14671701. Material requests should be addressed to Catherine Larzul catherine.larzul@inrae.fr, and Jordi Estellé jordi.estelle@inrae.fr or Claire Rogel-Gaillard claire.rogel-gaillard@inrae.fr.
