# Supplementary material for: Driving gut microbiota enterotypes through host genetics
Source: Microbiome. 2024 Jun 28;12:116. doi: 10.1186/s40168-024-01827-8 (PMC11214205; doi:10.1186/s40168-024-01827-8)
Supplement: Supplementary file 2 — Supplementary Material 1: Figure S1. Clustering of 60-day-old piglets from the G0 basal population into two enterotypes. (A) 313 G0 piglets comprising 86 piglets with varying enterotype classification during the clustering process over 100 iterations; (B) The subset of 227 piglets that do not change enterotype during the clustering process over 100 iterations. The subset of 15 females submitted to the shotgun metagenomics analysis were chosen from these two groups that are always categorized to the same enterotype. [file 40168_2024_1827_MOESM1_ESM.docx]

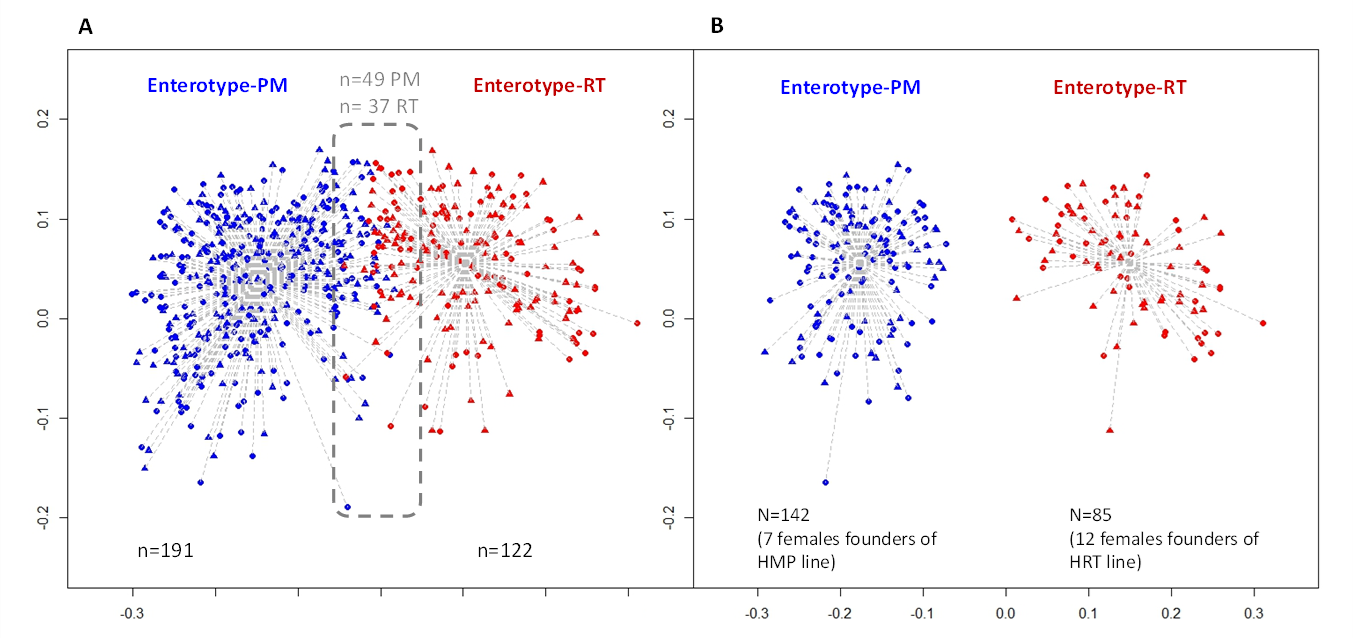


**Figure S1. Clustering of 60-day-old piglets from the G0 basal population into two enterotypes.** (A) 313 G0 piglets comprising 86 piglets with varying enterotype classification during the clustering process over 100 iterations; (B) The subset of 227 piglets that do not change enterotype during the clustering process over 100 iterations. The subset of 15 females submitted to the shotgun metagenomics analysis were chosen from these two groups that are always categorized to the same enterotype.
