# Supplementary material for: Driving gut microbiota enterotypes through host genetics
Source: Microbiome. 2024 Jun 28;12:116. doi: 10.1186/s40168-024-01827-8 (PMC11214205; doi:10.1186/s40168-024-01827-8)
Supplement: Supplementary file 6 — Supplementary Material 5: Figure S2. Individual variability of the taxonomic composition of each enterotype at the genus level on 60-day-old piglets from the G0 basal population. Only piglets that did not change enterotype during the classification process were represented on this figure. Barplots represent the relative abundance of the main genera observed in the communities (median relative abundance above 1.5 in at least one enterotype). [file 40168_2024_1827_MOESM5_ESM.docx]

**
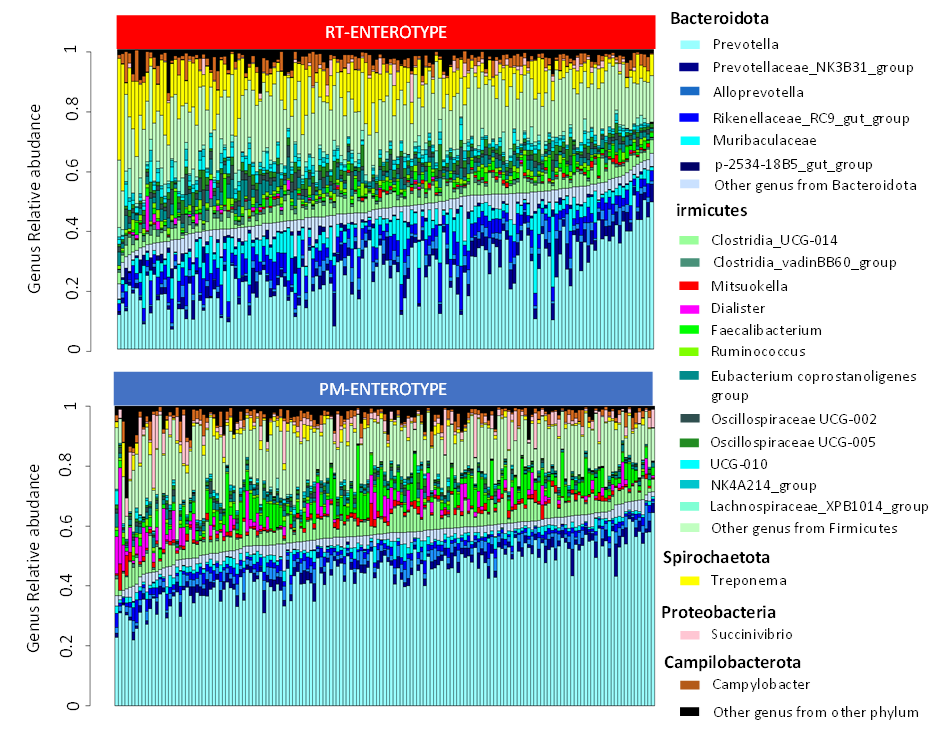
**

**Figure S2. Individual variability of the taxonomic composition of each enterotype at the genus level on 60-day-old piglets from the G0 basal population**. Only piglets that did not change enterotype during the classification process were represented on this figure. Barplots represent the genus relative abundance of the main genera observed in the communities (median relative abundance above 1.5 in at least one enterotype).
