# Supplementary material for: Driving gut microbiota enterotypes through host genetics
Source: Microbiome. 2024 Jun 28;12:116. doi: 10.1186/s40168-024-01827-8 (PMC11214205; doi:10.1186/s40168-024-01827-8)
Supplement: Supplementary file 7 — Supplementary Material 6: Figure S3. Differential abundances of a selection of MetaGenomics species (MGS) between PM and RT enterotypes. Abundance of the 12 most enriched MGS identified with shotgun metagenomics in either RT (A) or PM animals (B). Two subsets of 15 females are considered, blue and red boxplots corresponding to PM and RT animals respectively. Here, the abundance is the estimated genome sequencing coverage at similar sequencing depth. [file 40168_2024_1827_MOESM6_ESM.docx]

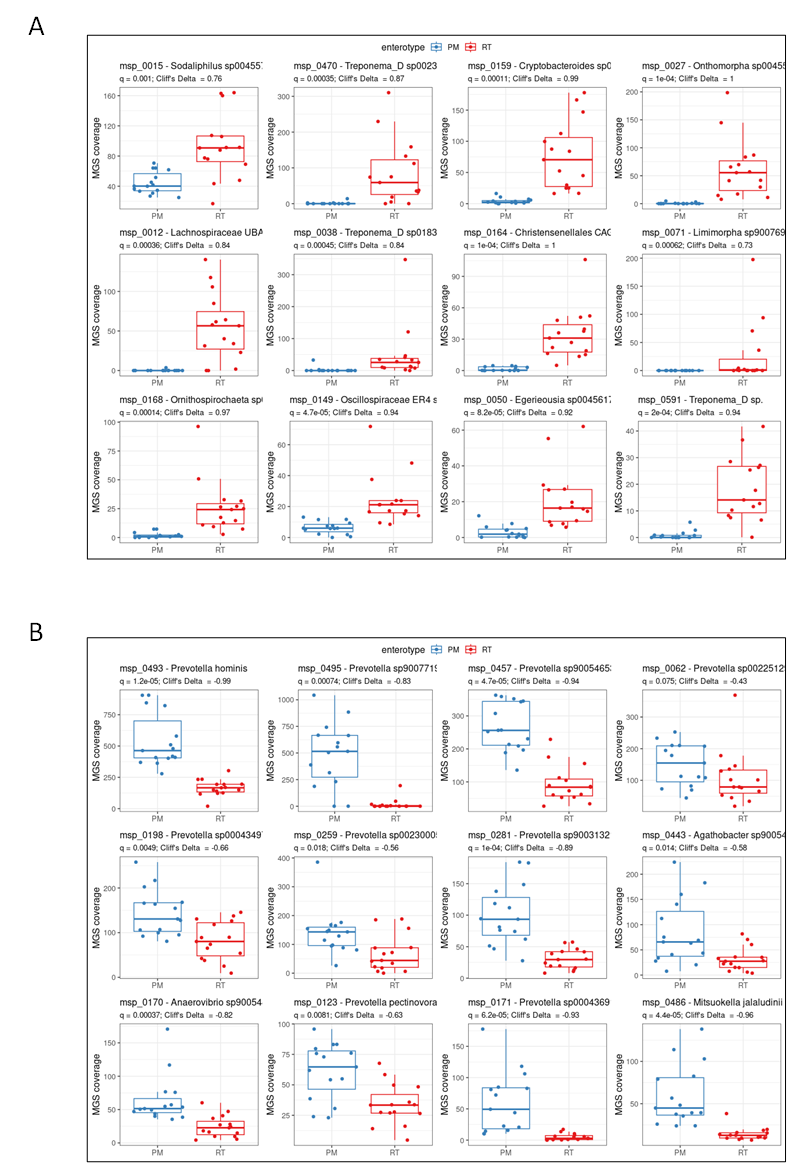


**Figure S3. Differential abundances of a selection of metagenomics species between PM and RT enterotypes**. Abundance of the 12 most enriched MetaGenomic Species identified with shotgun metagenomics in either RT (A) or PM animals (B). Two subsets of 15 females are considered, blue and red boxplots corresponding to PM and RT animals respectively. Here, the abundance is the estimated genome sequencing coverage at similar sequencing depth.
